# Early body composition outcomes of infants born very preterm and receiving high volume, human milk feedings (≥170 ml/kg/day) before postnatal day 14

**DOI:** 10.1038/s41372-025-02469-w

**Published:** 2025-10-31

**Authors:** Emily Gunawan, Madhuri Molleti, Ariel A. Salas

**Affiliations:** Division of Neonatology, Department of Pediatrics, University of Alabama at Birmingham, Birmingham, AL USA

**Keywords:** Outcomes research, Epidemiology

## Introduction

Several feeding strategies during the neonatal period aim to minimize postnatal weight loss, accelerate return to birthweight, and improve catch-up growth. Concerns exist, however, that rapid early growth in preterm infants could result in excessive fat mass accumulation [1].

While there is general consensus on the protective effects of human milk-based diets on body composition, even in the context of variability in growth rates [2], the optimal volume of human milk needed to support early growth and preserve body composition remains unclear. Recommendations for enteral feeding volumes in preterm infants range widely—from 140 to 200 ml/kg/day—and no consensus exist on the ideal maximum target volume [3, 4].

In a previous publication [1], we reported that very-low-birthweight infants who were fed either maternal milk or preterm formula and randomized to receive feeding volumes between 180 and 200 ml/kg/day had faster weight gains without significant differences in body fat percentage (%BF) at the time of hospital discharge. In this brief report, we evaluated the association between higher enteral feeding volumes ( ≥ 170 ml/kg/day) before postnatal day 14 and early growth and body composition outcomes in infants born very preterm, between 28 and 32 weeks of gestation.

## Materials/subjects and methods

This was a retrospective cohort study conducted at a tertiary neonatal unit at the University of Alabama at Birmingham (UAB) between 2020 and 2022. Infants with gestational ages between 28 and 32 weeks were included if parental consent had been obtained to perform a body composition assessment around postnatal day 14. Infants with congenital anomalies affecting growth were excluded. All infants included received enteral nutrition with maternal or donor milk starting at volumes ranging between 20 and 80 ml/kg/day within the first 36 h after birth and achieved enteral feeding volumes of 150 ml/kg/day or more by postnatal day 14. Infants who achieved enteral feeding volumes ≥ 170 ml/kg/day by postnatal day 14 were assigned to the high-volume group. Infants who achieved enteral feeding volumes <170 ml/kg/day by postnatal day 14 were assigned to the usual-volume group.

The primary outcome was %BF measured using air-displacement plethysmography around postnatal day 14. Secondary outcomes were time to regain birthweight and change in weight-for-age z scores from birth to 14 days.

Statistical analyses were performed using SAS 9.4 (SAS Institute Inc., Cary, NC).

## Results

A total of 212 infants were included in the study. Birthweight did not differ between groups (Table 1). Both groups reached full enteral feeding ( >150 ml/kg/day) at a median of 7 days (*p* = 0.11). In the high-volume group, the median postnatal age at reaching enteral feeding volumes ≥170 ml/kg/day was 10 days (IQR: 8–12) while the median duration of enteral feeding volumes ≥170 ml/kg/day in the first 14 days was 4 days (IQR: 3–6).

**Table 1:** Baseline characteristics and outcomes.

	High-volume group (*n* = 106)	Usual-volume group (*n* = 106)	*p* value^a^
Demographic characteristics			
Birth weight in grams, mean ± SD	1450 ± 282	1466 ± 297	0.79
Gestational age in weeks, median (IQR)	31 (30–32)	30 (29–32)	0.03
Small for gestational age, n (%)	2 (2)	3 (3)	0.65
Weight-for-age z score, mean ± SD	−0.32 ± 0.72	−0.06 ± 0.79	0.007
Male, n (%)	52 (49)	48 (45)	0.58
Black race, n (%)	46 (43)	47 (44)	0.89
Year			
2020–2021	29 (27)	53 (50)	0.0007
2022–2023	77 (73)	53 (50)	
Postnatal age in days at the time of initiation of enteral feedings, median (IQR)	1 (1–1)	1 (1–2)	0.96
Postnatal age in days at the time of establishment of full enteral nutrition ( >150 ml/kg/day), median (IQR)	7 (5–8)	7 (5–9)	0.11
Postnatal age in days at the time of initiation of human milk fortification, median (IQR)	11 (8–13)	12 (9–13)	0.22
Proportion of infants receiving enteral feedings volumes > 60 ml/kg/day within the first 36 h after birth, n (%)	58 (55)	43 (41)	0.04
Fat mass in kg at 14 days, median (IQR)	0.11 (0.06–0.18)	0.09 (0.05–0.15)	0.15
%BF at 14 days, median (IQR)	7.3 (4.0–10.2)	6.1 (3.6–8.6)	0.12
Fat mass z score at 14 days, mean ± SD	−0.67 ± 0.92	−0.76 ± 0.88	0.51
%BF z score at 14 days, mean ± SD	−0.15 ± 1.05	−0.31 ± 1.04	0.33
Fat-free mass in kg, mean ± SD	1.50 ± 0.27	1.49 ± 0.26	0.72
Fat-free mass z score, mean ± SD	−1.66 ± 1.07	−1.37 ± 0.98	0.08
Time to regain birthweight in days, median (IQR)	14 (12–16)	15 (12–17)	0.02
Change in weight z scores from birth to 14 days, mean ± SD	−0.95 ± 0.28	−1.04 ± 0.32	0.03
Difference between actual and target weight at term-equivalent age or discharge in grams, median (IQR)^b^	−29 (−134 to 61)	−52 (−174 to 67)	0.32

^a^Derived by using Wilcoxon’s rank-sum test. *T* test, or Chi-square test.

^b^Determined by using an online resource that generates an individualized growth “trajectory” in preterm infants (www.growthcalculator.org).

**Fig. 1:**
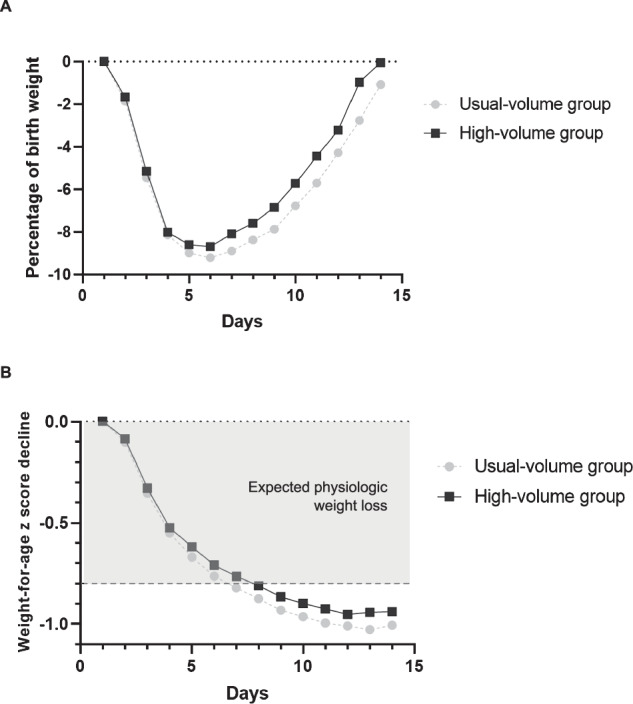
Weight loss patterns in preterm infants. Weight loss during the first 14 days in infants born very preterm using (**A**) birth weight percentages and **B** declines in weight z scores. The figure displays unadjusted means by groups using unadjusted repeated measures mixed models.

There were no significant differences in %BF around postnatal day 14 between the two groups (Table 1). Infants in the high-volume group had an earlier return to birthweight (14 vs 15 days; *p* = 0.02) compared to the usual-volume group (Fig. 1). Smaller declines in weight z scores from birth to postnatal day 14 were observed in the high-volume group (−0.95 ± 0.28 vs −1.04 ± 0.32; *p* = 0.03).

## Discussion

In this cohort of infants born very preterm, higher enteral feeding volumes during the first 14 days were not associated with higher %BF at around postnatal day 14. The weight loss patterns observed in the high-volume group suggest that advancing enteral feed volumes to ≥170 ml/kg/day within the first two weeks may confer modest growth benefits without adverse effects on early body composition, but the clinical significance of these results remains uncertain without longitudinal data. Our findings also support the safety and potential utility of higher-volume enteral feeding strategies in clinically stable preterm infants receiving predominantly human milk-based diets [5].

The main limitations of this study are the observational design, which cannot rule out the possibility that baseline differences in clinical stability existed between groups, and the lack of consistency in feeding practices during the study period. Although advancing volumes up to 200 ml/kg/day remained at the discretion of individual clinicians throughout the study period, it was not formally recommended until late 2021. From 2022 onward, fortification to 24 kcal/oz was typically initiated between postnatal days 7 and 14. In 2020 and early 2021, the standard approach was to start enteral feeds at 20–30 ml/kg/day, while in late 2021, as part of clinical trial participation, many infants received initial feeds of up to 80 ml/kg/day. Nonetheless, an important strength of this study is that it underscores the potential value of standardizing early body composition assessments by postnatal day rather than postmenstrual age. This approach may be particularly relevant for clinically stable infants who are discharged earlier before catch-up growth occurs.

Further prospective studies are needed to evaluate the long-term implications of high-volume feeding on growth, neurodevelopment, and metabolic health, and to identify which subgroups of preterm infants may benefit most from this approach.

## Data Availability

De-identified individual participant data will be made available upon publication to researchers who provide a methodologically sound proposal for use in achieving the goals of the approved proposal. Further inquiries can be directed to the corresponding author.
